# Determining the IgG concentrations in bovine colostrum and calf sera with a novel enzymatic assay

**DOI:** 10.1186/s40104-018-0287-4

**Published:** 2018-08-27

**Authors:** M. Drikic, C. Windeyer, S. Olsen, Y. Fu, L. Doepel, J. De Buck

**Affiliations:** Department Production Animal Health, Faculty of Veterinary Medicine, University of Calgary, Calgary, AB Canada

**Keywords:** Cattle, Colostrum, Immunoglobulins, Passive immunity, Radial immunodiffusion, Split trehalase

## Abstract

**Background:**

Immune protection in newborn calves relies on a combination of the timing, volume and quality of colostrum consumed by the calf after birth. Poor quality colostrum with inadequate immunoglobulin concentration contributes to failed transfer of passive immunity in calves, leading to higher calf morbidity and mortality. Therefore, estimating colostrum quality and ensuring the transfer of passive immunity on farm is of critical importance. Currently, there are no on-farm tools that directly measure immunoglobulin content in colostrum or serum. The aim of this study was to apply a novel molecular assay, split trehalase immunoglobulin G assay (STIGA), to directly estimate immunoglobulin content in dairy and beef colostrum and calf sera, and to examine its potential to be developed as on-farm test. The STIGA is based on a split version of trehalase TreA, an enzyme that converts trehalose into glucose, enabling the use of a common glucometer for signal detection. In a first study, 60 dairy and 64 beef colostrum and 83 dairy and 84 beef calf sera samples were tested with STIGA, and the resulting glucose production was measured and compared with radial immunodiffusion, the standard method for measuring immunoglobulin concentrations.

**Results:**

Pearson correlation coefficients between the methods were determined and the sensitivity, specificity, and accuracy of the test were calculated for different colostrum quality and failed transfer of passive immunity cut-off points. The correlations of the STIGA measured by colorimetric enzymatic reaction compared to radial immunodiffusion for dairy and beef colostrum were 0.72 and 0.73, respectively, whereas the correlations for dairy and beef sera were 0.9 and 0.85, respectively. Next, STIGA was tested in a blinded study with fresh colostrum and serum samples where the correlation coefficient was 0.93 and 0.94, respectively. Furthermore, the performance of STIGA followed by glucometer readings resulted in correlations with radial immunodiffusion of 0.7 and 0.85 for dairy and beef colostrum and 0.94 and 0.83 for dairy and beef calf serum.

**Conclusions:**

A split TreA assay was validated for measurement of the immunoglobulin content of colostrum and calf sera using both a lab-based format and in a more user-friendly format compatible with on-farm testing.

## Background

Survival and wellbeing of the newborn calf is strongly dependent on the transfer of immunoglobulins G (IgG) from the colostrum consumed immediately after birth [1]. If the consumed colostrum does not contain sufficient IgG (IgG content > 50 mg/mL for dairy colostrum and > 100 mg/mL for beef colostrum [1–3]), the calf will likely suffer from failed transfer of passive immunity (FTPI) (IgG content < 10 mg/mL for dairy calves [2, 4] and < 24 mg/mL for beef calves [5, 6]). The consequence is a higher risk of mortality and morbidity [1]. With almost 30% [7] of dairy colostrum samples being poor quality and FTPI occurring in 37.1% of calves [8], farmers must be encouraged to measure CQ before feeding the colostrum to their calves. To achieve this goal, it is important to offer them a testing method that is user-friendly, fast, accurate, and inexpensive. Currently, there are several on-farm methods used to estimate IgG content in bovine colostrum and calf serum. Most of them have the disadvantage of estimating IgG concentration indirectly, which can lead to the overestimation of IgG concentration [7, 9]. Radial immunodiffusion (RID) is considered the gold standard [10, 11] but cannot be performed in the field and is slow and costly.

**Fig. 1:**
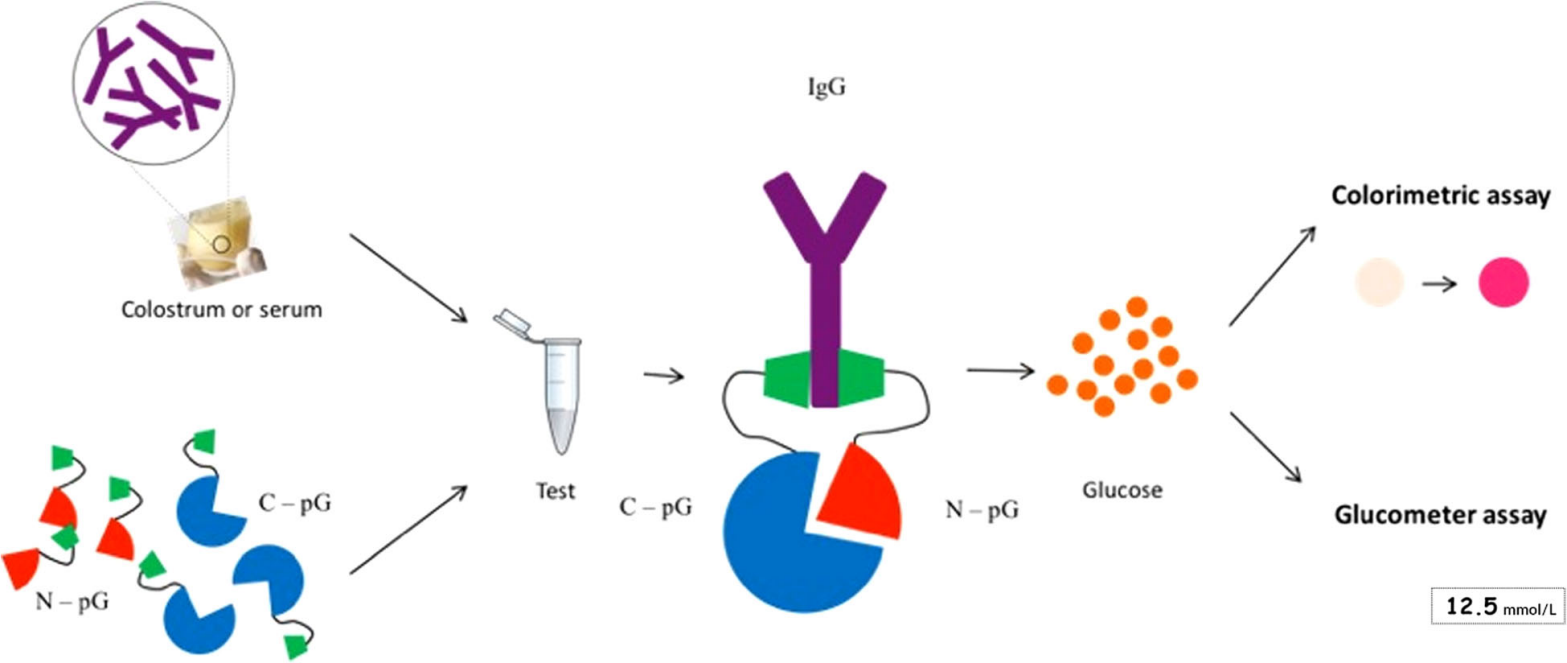
Schematic representation of the STIGA detection mechanism used to estimate the quantity of IgG in colostrum or serum

A novel split trehalase IgG quantification assay (STIGA) was developed by our group [12] and is compatible with on-farm application. The STIGA is based on the glycolytic enzyme, trehalase (TreA), which converts trehalose into glucose. TreA is split in two non-functional fragments [13], TreA^N^ (N) and TreA^C^ (C), both fused to streptococcal protein G (pG) [14]. Protein G binds specifically to the constant region of IgG (Fc) and acts as a sensor for IgG independent of antigen binding specificity. When the two fusion proteins are incubated with samples containing IgG (e.g. colostrum or serum), they both bind to the Fc of IgG, leading to the dimerization and re-activation of TreA. Re-activated TreA produces glucose from trehalose, which can be detected by a colorimetric assay (STIGA, Fig. 1) or by glucometer (STIGA^FIELD^; Fig. 1).

The aim of this study was to use STIGA to directly estimate IgG concentration in beef and dairy colostrum samples and in calf serum samples, to compare its performance to RID and to examine its potential to be developed as on-farm test.

## Methods

### Sample collection

Dairy colostrum (*n* = 60) and serum samples (*n* = 83) were randomly selected from samples collected for a previous study [7]. Colostrum samples were collected from 13 dairy farms in central Alberta between February and July of 2012. Samples were collected by the farm owners, frozen at − 20 °C and transported to the University of Calgary where they were stored at − 80 °C. Serum samples were collected during the same period by research personnel from bull and heifer calves > 24 h and ≤ 8 d of age. The samples were stored on ice and transported to the University of Calgary where the serum was harvested by centrifugation at 1,800×*g* at 4 °C for 25 min. Serum samples were also stored at − 80 °C. Beef colostrum (*n* = 64) and serum samples (*n* = 84) were collected during calving seasons (January through May) from 2014 to 2016 from six privately-owned cow-calf operations in Alberta. Beef colostrum and blood samples were processed according to the same protocol as the dairy samples. The IgG concentrations in the dairy colostrum and serum samples were determined by RID by Prairie Diagnostic Laboratories (University of Saskatchewan, Saskatoon, SK) while IgG content in the beef colostrum and serum samples was determined by the Quality Assurance Laboratory of the Saskatoon Colostrum Company Ltd. (SCCL, University of Saskatchewan, Saskatoon, SK) by RID. Dairy colostrum (*n* = 20) and calf serum samples (*n* = 25) used in the blinded study were obtained from the Quality Assurance Laboratory of SCCL where they were randomly selected and tested by RID for this purpose.

### Strains, plasmids, and other materials

Purified bovine IgG (12.8 mg/mL) used for standard curves was purchased from Sigma-Aldrich (Oakville, Canada). Plasmids used in this work were described previously [12]. In short, the gene coding for *E. coli* glycolytic enzyme TreA [13] was split in two fragments, *TreA*^*N*^ (198 bp long) and *TreA*^*C*^ (1,368 bp long) through PCR amplification. Each gene fragment was fused with the coding sequence of protein G [14] (residues: 270–324; AC: P19909) and cloned in pETDuet expression vector (Novagen; Oakville, Canada) using NcoI and AvrII restriction sites.

### Protein purification and lyophilisation

Proteins were recombinantly expressed in BL-21 ΔTreA strain and purified on Ni-NTA resin as previously described [12]. Briefly, BL-21 ΔTreA cultures were induced with 0.5 mmol/L of IPTG (isopropil-β-D-1-tiogalattopiranoside) (UBP Bio; Aurora, Colorado, USA) and harvested after 3 h at 37 °C. Bacterial pellets with recombinant proteins were resuspended in 6 mol/L guanidinium buffer, sonicated and loaded on equilibrated Ni-NTA resin (Thermo-Fisher Scientific; Ottawa, Canada). Proteins were refolded on resin during washing steps containing gradually decreasing guanidinium-HCl concentrations and eluted in Elution buffer containing 250 mmol/L of Imidazole. Protein preparations used in the blinded study were purified using AKTA pure system (GE Healthcare Life sciences, Pittsburgh, Pennsylvania, USA). Briefly, 0.5 L of recombinant cultures were induced, lysed as described previously. After lysis, the samples were resuspended in 40 mL of denaturing resuspension buffer, loaded on HisTrap HP 1 mL column (GE Healthcare Life sciences, Pittsburgh, Pennsylvania, USA), refolded on column by continuously decreasing concentration of guanidinium-HCl during the wash step and eluded in native elution buffer (0.5 mol/L imidazole).

Finally, all protein preparations were dialyzed against 1 L of sodium maleate buffer (50 mmol/L, pH 6) with Snakeskin (Thermo-Fisher Scientific; Ottawa, Canada) for 24 h at 25 °C and protein concentration was determined by Qubit assay (Thermo-Fisher Scientific; Ottawa, Canada). Lyophilized reagents were prepared as described previously [12]. Briefly, proteins were mixed in a1 to 1 weight ratio with BSA, frozen in microtiter plate wells at − 80 °C and then lyophilized over night at − 85 °C and 200 mT.

### Split trehalase immunoglobulin G assay (STIGA)

The STIGA quantification assays were performed in two ways. Glucose measurements were determined with a GOx-HRP colorimetric assay for all samples (STIGA) and a sample subset chosen randomly (dairy colostrum *n* = 14; beef colostrum *n* = 14; dairy calf sera *n* = 18; and beef calf sera *n* = 18) was analyzed with a glucometer test strip based assay (STIGA^FIELD^).

GOx-HRP colorimetric STIGA: Colostrum and calf serum samples were diluted in sodium maleate buffer (50 mmol/L, pH 6) (dairy colostrum 1 to 2,000; beef colostrum 1 to 4,000; dairy and beef calf serum 1 to 1,000). STIGA was performed with 20 μg of C-pG and 5.2 μg of N-pG (1 to 1 mole ratio) for colostrum or 10 μg of C-pG and 2.6 μg of N-pG for serum in sodium maleate buffer with 250 mmol/L of trehalose (Sigma-Aldrich; Oakville, Canada) in a final volume of 150 μL. In the blinded study, STIGA was performed with small modifications: 5 μg of of C-pG and 1.3 μg of N-pG were used and both colostrum and serum were diluted 1 to 1,000 in sodium maleate buffer. The glucose concentration was measured using glucose oxidase form *Aspergillus niger* (2.6 U/mL; Sigma Aldrich; Oakville, Canada), horseradish peroxidase (0.2 U/mL; Sigma-Aldrich; Oakville, Canada), and O-dianisidine (0.5 mmol/L; Sigma-Aldrich; Oakville, Canada) in sodium maleate buffer (50 mmol/L, pH 6). Absorbance (optical density; OD) was measured every minute for 90 min with an EnSpire multimode plate reader at 450 nm (PerkinElmer; Waltham, Massachusetts, USA).

Glucose test strips based STIGA (STIGA^**FIELD**^): This assay was performed with lyophilized protein preparations. Lyophilized proteins were resuspended in 150 μL of sodium-maleate buffer with 250 mmol/L of trehalose containing the same colostrum or serum dilution used in previous assay. Glucose production was measured by Accu-Chek Aviva Blood Glucometer (Roche; Mississauga, Canada) and test strips after 90 min.

### Statistical analysis

GOx-HRP colorimetric STIGA: The IgG content of dairy and beef colostrum and sera analyzed with the STIGA was correlated with RID-determined IgG concentration using Pearson correlation coefficients in Excel (Microsoft). Sensitivity, specificity, positive (PPV) and negative predicted values (NPV) and accuracy were calculated for different OD cut-off points. The threshold for adequate quality colostrum was set at 50 mg/mL [1–3] for dairy colostrum and at 100 mg/mL for beef colostrum. Because there is no reported established cut-off for beef colostrum, the value of 100 mg/mL was used based on the equation: adequate colostrum concentration (mg/mL) = [24 mg/mL (adequate passive immunity) × 3.8 L (plasma volume for average birth weight of 42 kg)] ÷ [0.3 (estimated efficiency of absorption) × 3 L (estimated volume of colostrum consumed in 24 h)]. Sensitivity, specificity, PPV, NPV, and accuracy were calculated using RID as gold standard with the threshold for FTPI for dairy calves at 10 mg/mL for dairy calves [2, 4] and at 24 mg/mL for beef calves [5, 6]. Four colostrum and four serum samples were run in triplicates on two different days to calculate intra-assay CV% and inter-assay CV%.

Glucose test strips based STIGA (STIGA^FIELD^): The colostrum and sera IgG values determined by STIGA^FIELD^ were correlated with RID determined IgG concentrations by Pearson coefficient correlation.

## Results

### Detection of IgG in bovine colostrum

**Fig. 2:**
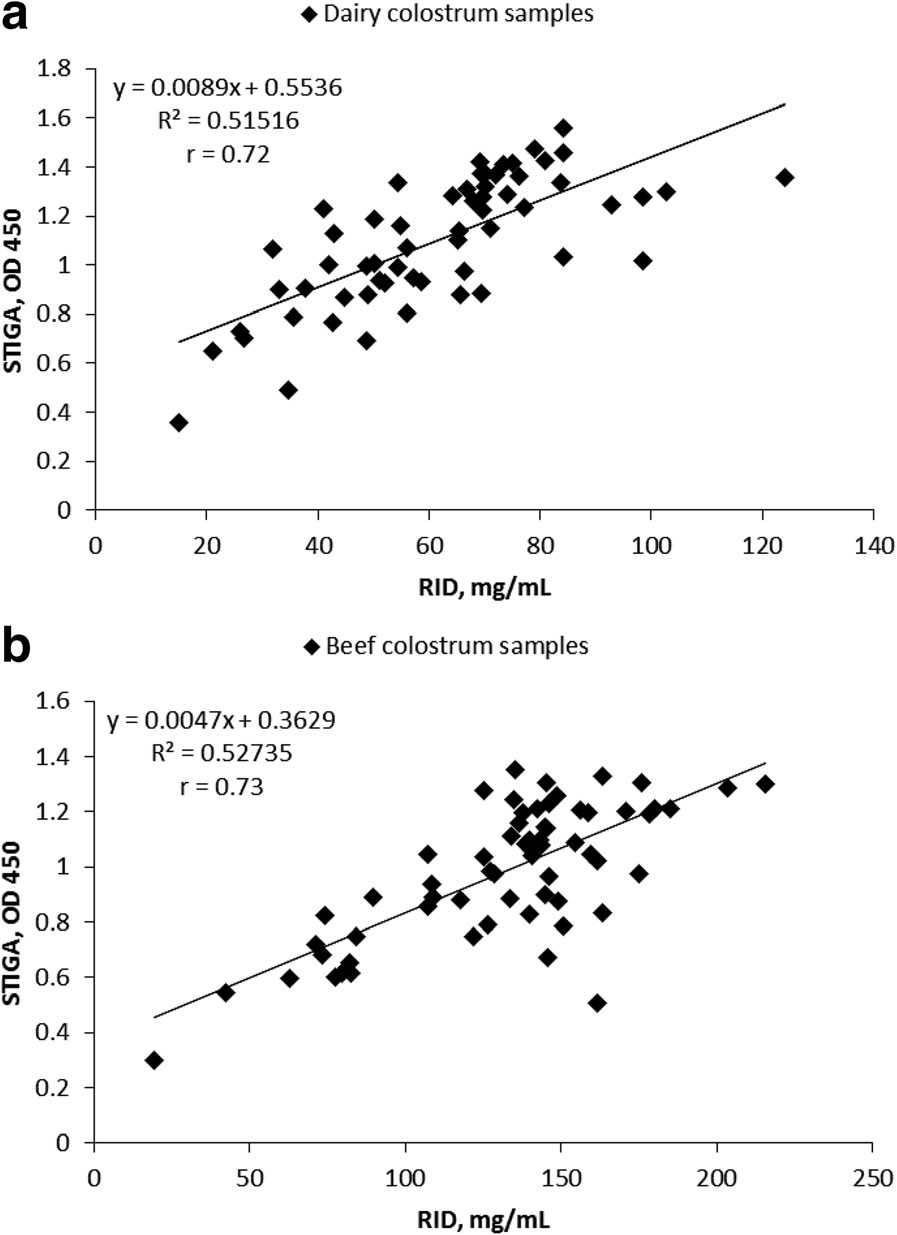
Correlation between RID determined IgG concentrations in colostrum and glucose levels determined by STIGA. Scatter plot comparing IgG concentration measured by STIGA (OD 450) and by RID (mg/mL) in: **a**, dairy colostrum (*n* = 60); **b**, beef colostrum (*n* = 64)

The Pearson correlation coefficient between STIGA and RID for dairy colostrum was 0.72 (Fig. 2a), whereas for beef colostrum the correlation coefficient was 0.73 (Fig. 2b). With dairy colostrum samples, STIGA had the highest sensitivity (64.7%) and specificity (93%) when an OD of 0.9 was used as the cut-off (Table 1). Test positive (poor quality) dairy samples had a 71.4% chance of being truly poor quality (i.e., PPV) and test negative (adequate quality) samples had an 84.8% chance of being truly adequate (i.e., NPV). STIGA identified 23% of analyzed colostrum samples to be of poor quality whereas RID identified 28.3% of samples as poor quality.

**Table 1:** Diagnostic test characteristics for STIGA in colostrum

Cut point, OD 450	Accuracy,%	Se, %	Sp, %	PPV, %	NPV, %
Dairy colostrum					
0.6	75.0	11.8	100.0	100.0	74.1
0.7	78.3	23.5	100.0	100.0	76.8
0.8	85.0	47.1	100.0	100.0	82.7
0.9	85.0	64.7	93.0	78.6	87.0
1.0	78.3	76.5	79.1	59.1	89.5
1.1	75.0	88.2	69.8	53.6	93.8
1.2	68.3	94.1	58.1	47.1	96.2
Beef colostrum					
0.6	84.4	25.0	98.1	75.0	85.0
0.7	90.6	66.7	96.2	80.0	92.6
0.8	89.1	83.3	90.4	66.7	95.9
0.9	79.7	100.0	75.0	48.0	100.0
1.0	71.9	100.0	65.4	40.0	100.0
1.1	53.1	100.0	42.3	28.6	100.0
1.2	42.2	100.0	28.8	24.5	100.0

Accuracy, sensitivity (Se), specificity (Sp), positive predictive value (PPV) and negative predictive value (NPV) calculated for OD cut points for detecting adequate quality colostrum with STIGA with cut-offs of 50 mg/mL IgG for dairy colostrum and 100 mg/mL of beef colostrum determined by RID; for dairy colostrum (*n* = 60) and beef colostrum(*n* = 64)

For beef colostrum, STIGA reached its highest sensitivity (83.3%) and specificity (90.3%) when an OD of 0.8 was used as the cut-off value (Table 2.). At this cut-off, the beef colostrum had a PPV of 66.7% and a NPV of 95.9%. STIGA identified 23.4% of colostrum samples as poor quality, whereas with RID 18.8% of samples were poor quality.

**Table 2:** Diagnostic test characteristics for STIGA in calf serum

Cut point, OD 450	Accuracy, %	Se, %	Sp, %	PPV, %	NPV, %
Dairy calf serum					
0.2	91.6	77.8	98.2	95.5	90.2
0.3	86.7	100.0	80.4	71.1	100.0
0.4	67.5	100.0	51.8	50.0	100.0
0.5	55.4	100.0	33.9	42.2	100.0
0.6	45.8	100.0	19.6	37.5	100.0
0.7	41.0	100.0	12.5	35.5	100.0
0.8	34.9	100.0	3.6	33.3	100.0
Beef calf serum					
0.1	85.7	7.7	100.0	100.0	85.5
0.2	90.5	38.5	100.0	100.0	102.9
0.3	92.9	69.2	97.2	81.8	94.5
0.4	83.9	100.0	91.5	46.4	116.1
0.5	52.4	100.0	43.7	24.5	100.0
0.6	31.0	100.0	18.3	18.3	100.0
0.7	16.7	100.0	1.4	15.7	100.0

Accuracy, sensitivity (Se), specificity (Sp), positive predictive value (PPV), and negative predictive value (NPV) calculated for OD cut points for detecting FTPI with STIGA with cut-offs of 10 mg/mL IgG for dairy calf sera and 24 mg/mL for beef calf sera determined by RID; for dairy calf sera (*n* = 83) and beef calf sera (*n* = 84)

In both sample groups the background glucose was undetectable prior to addition of STIGA reagents (data not shown).

### Detection of IgG in calf serum

**Fig. 3:**
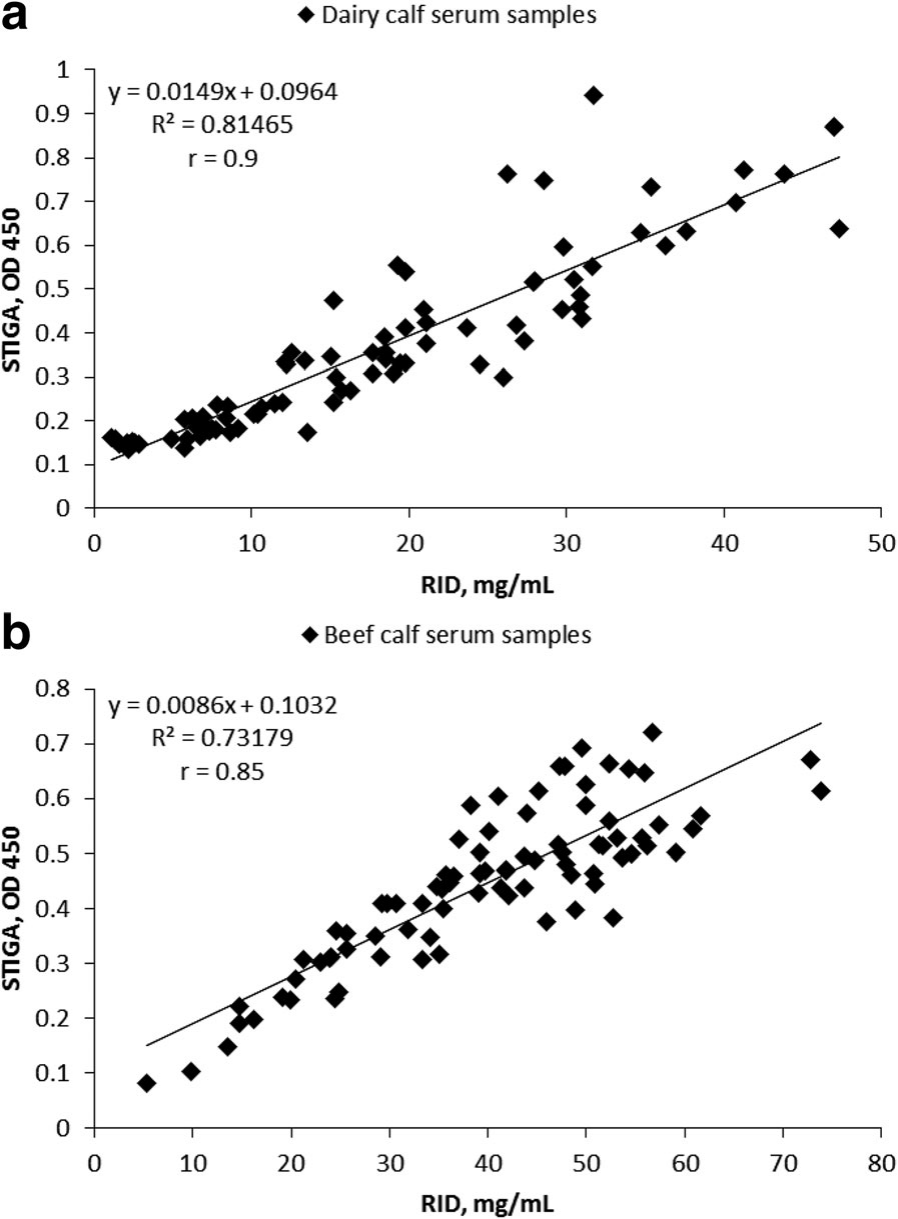
Correlation between RID determined IgG concentrations in serum and glucose levels determined by STIGA. Scatter plot comparing IgG concentrations determined by RID (mg/mL) and by STIGA (determined as optical density OD 450) in: **a**, dairy calf sera (*n* = 83); **b**, beef calf sera (*n* = 84)

The Pearson correlation coefficient between serum RID-determined IgG and STIGA-determined IgG was 0.9 for dairy samples (Fig. 3a) and 0.85 for beef samples (Fig. 3b). The highest sensitivity (77.8%) and specificity (98.2%) by STIGA was reached at an OD of 0.2 (Table 2) in dairy calf serum samples. With beef calf sera, the highest sensitivity (69.2%) and specificity (97.2%) were reached at an OD of 0.3 (Table 2). In dairy calf serum samples, STIGA had a 95% chance of correctly identifying FTPI and 90% chance of correctly showing that the transfer of passive immunity had occurred whereas in beef calf serum samples, STIGA had 75% and 94.5% chance of correctly identifying FTPI or successful transfer of passive immunity, respectively. FTPI was observed in 26.5% of dairy calf serum samples and 13% of beef calf serum samples by STIGA, compared with 32.5% and 15.5%, respectively, diagnosed by RID.

In both sample groups the background glucose was undetectable prior to addition of STIGA reagents (data not shown).

### Detection of IgG by STIGA in samples of unknown IgG concentration (blinded study)

**Fig. 4:**
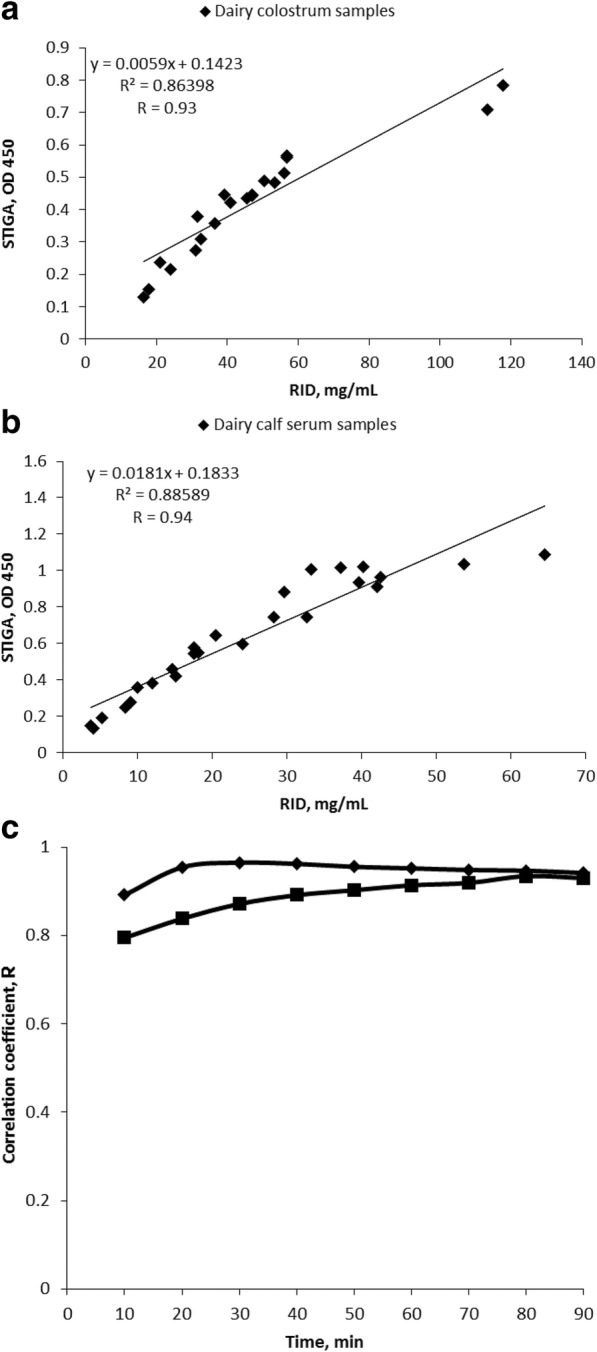
Correlation between RID determined IgG concentrations in colostrum and serum and glucose levels determined by STIGA in blind study. Scatter plot comparing IgG concentrations determined by RID (mg/mL) and by STIGA (determined as optical density OD 450) in blinded study: **a**, dairy colostrum (*n* = 20); **b**, dairy calf sera (*n* = 25); **c**, correlation coefficient between RID (mg/mL) and STIGA (OD 450) during the assay (squares – colostrum samples; diamonds – serum samples)

STIGA was used to detect total IgG content in fresh dairy colostrum and serum samples (recent samples that were frozen only once) of IgG concentrations unknown to the researchers. The concentration obtained by STIGA was correlated with RID and the obtained correlation coefficients were 0.93 for colostrum samples and 0.94 for serum samples (Fig. 4a and b). High correlation values (> 0.8 for colostrum and > 0.9 for sera) were observed after 10 min of run time (Fig. 4c and d). STIGA reached the highest sensitivity (100%) and specificity (71.4%) for detecting good quality colostrum at an OD of 0.5 (Table 3), while it reached the highest sensitivity (100%) and specificity (94.7%) for detecting FTPI at an OD of 0.4 (Table 3). STIGA was able to correctly identify poor quality colostrum (i.e. PPV) or adequate quality colostrum (i.e. NPV) in 86.8% or 100% of cases, respectively. In serum samples STIGA had a PPV of 85.7% and NPV of 100%, respectively.

**Table 3:** Diagnostic test characteristics for STIGA in blind study

	Cut point, OD 450	Accuracy, %	Se, %	Sp, %	PPV, %	NPV, %
Colostrum	0.5	90	100	71.43	86.67	100
Serum	0.4	96	100	94.73	85.71	100

Accuracy, sensitivity (Se), specificity (Sp), positive predictive value (PPV), and negative predictive value (NPV) calculated for OD cut point for detecting adequate quality colostrum with STIGA with cut-offs of 50 mg/mL IgG for dairy colostrum and for detecting FTPI with STIGA with cut-offs of 10 mg/mL IgG for dairy calf sera determined by RID; for dairy colostrum (*n* = 25) and dairy calf sera (*n* = 20)

Four colostrum and four serum samples with different IgG concentrations were analyzed to calculate inter and intra assay variability. Intra and inter assay variability for colostrum samples were 3.5% and 11.5%, and for the serum samples, 2.5% and 8.1%, respectively.

### Detection of IgGs by STIGA using a glucometer (STIGA^FIELD^)

**Fig. 5:**
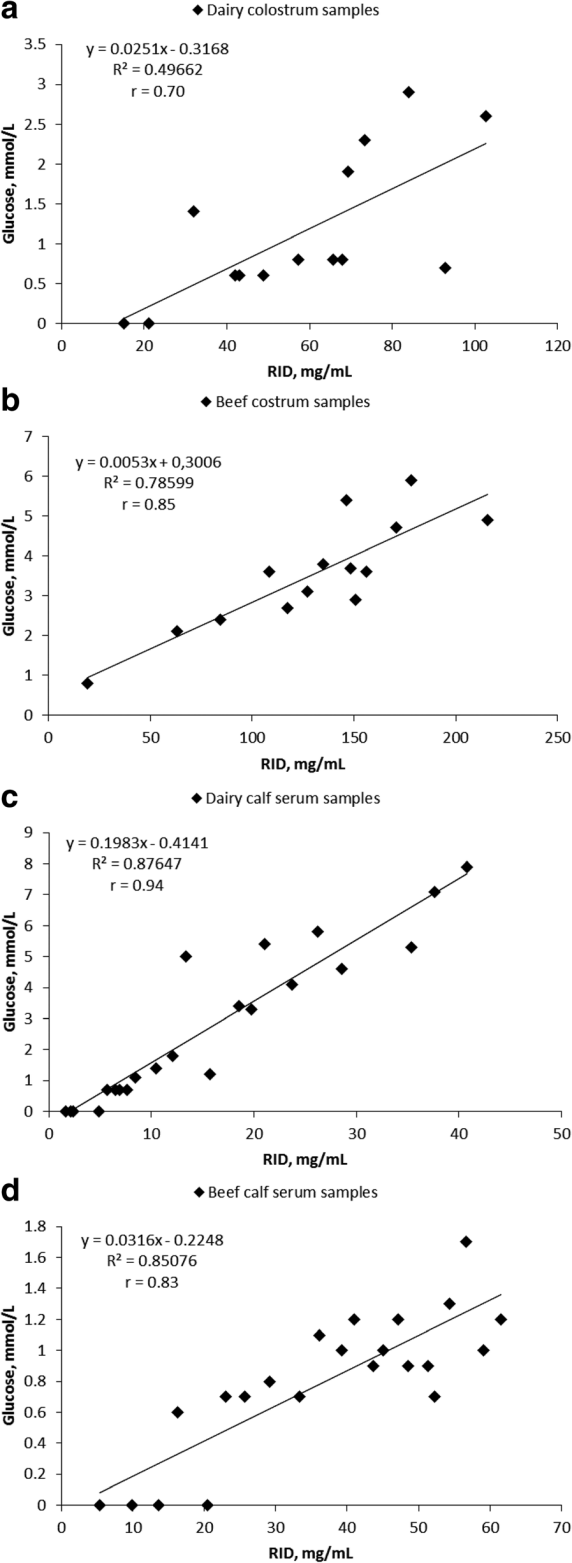
Correlation between RID determined IgG concentrations in colostrum and serum and glucose levels determined by STIGA^FIELD^ Scatter plots of IgG concentration measured by STIGA^FIELD^ (mmol/L of glucose measured by glucometer) and concentration determined by RID (mg/mL) for: **a**, dairy colostrum samples (*n* = 14); **b**, beef colostrum samples (*n* = 14); **c**, dairy calf serum samples (*n* = 22); **d**) beef calf serum samples (*n* = 22)

In order to evaluate STIGA for future on farm use, a smaller sample set for each test group was analyzed with an assay variant, STIGA^FIELD^. The correlation coefficients between RID determined IgG concentrations and glucose levels determined by STIGA^FIELD^ were 0.7 for dairy colostrum, 0.85 for beef colostrum, 0.94 for dairy calf sera, and 0.83 for beef calf sera (Fig. 5).

## Discussion

In this study, we described a novel assay named STIGA to measure the IgG concentration in bovine colostrum and serum. In contrast to the majority of assays developed for this purpose, STIGA is able to directly measure IgGs mediated by specific interaction of the STIGA reagents with IgGs.

Currently, there are several methods used to estimate IgG content in bovine colostrum and calf serum. Radial immunodiffusion estimates IgG concentration directly and, currently, is considered the gold standard for both colostrum and calf serum [11]. However, RID is a lab-based test that requires expensive reagents and long processing times, making it unsuitable for on-farm use. The colostrometer and Brix refractometer (optical or digital) are two devices most commonly used on farms to access colostrum quality [15–19]. Colostrometers measure the specific density of colostrum whereas the Brix refractometer measures its refraction index. Both of these methods have the disadvantage of estimating IgG concentration indirectly by measuring colostrum properties that are proxies for IgG concentration. These methods can lead to the overestimation of IgG concentration [7, 9]. Apart from these, there have been a number of other techniques explored including infrared spectroscopy (IR) [9] and immunoassays (IM) [20]. Although more sensitive than other assays, IR still requires expensive equipment and analysis of the acquired data [9], whereas the disadvantage of IM is that they only provide positive or negative results without any quantification of IgG [20]. For determination of IgG in calf sera, several tests are available such as sodium sulphate and zinc sulphate turbidity tests, immunoassays, measurement of g-glutamyl transferase activity, glutaraldehyde coagulation tests, and Brix refractometry [4, 21]. Apart from the immunoassays [22], including ELISA, these tests also have the disadvantage of not measuring IgG directly. Furthermore, many of them have poor sensitivity and specificity and require a lab setting and skilled personnel to perform the test.

Compared with the two most frequently used methods to analyse dairy colostrum, colostrometer and Brix, STIGA had the same level of agreement with RID, comparable sensitivity and improved specificity [7]. Applied to serum, STIGA has a high correlation with RID, as well as high sensitivity and specificity. Furthermore, when tested with fresh colostrum and serum samples of unknown concentrations, STIGA reached much higher level of agreement with RID then has been reported for Colostrometer or Brix assay [7]. Its sensitivity, specificity, PPV and NPV were improved as well. Furthermore, inter and intra assay variability were within the generally expected range [23]. The correlation value was high after the first 10 min, indicating that assay time could be much shorter than used in this study.

Moreover, direct measurement of the IgG offered by STIGA and RID overcomes many limitations and inaccuracies that arise from indirect determination of IgG. For example, colostrometer measurements are temperature dependent. Hence, if samples are not at room temperature (22 °C) when analysed, their IgG content will not be correctly determined. The measurements obtained by the Brix method, although more reliable than the colostrometer, measures total solid content to estimate IgG content and can be influenced by factors like nutrition that change the total solid but not the IgG concentration [24, 25]. Similarly, hydration status of the calves and type of colostrum used for first feed influence the total protein in calf serum. The Immunoassay kit (Colostrum bovine IgG quick test kit, Midlands Bio-Products, Boone, Iowa, USA) [20, 22] is the only on-farm method that directly measures IgG in colostrum or serum, but this test has the major disadvantage of not providing information about the actual concentration of the IgG in the sample but rather a YES or NO answer if the cut-off point was reached or not. While reaching the cut-off point might be sufficient to establish if FTPI occurred, this assumes the cut-point used by the assay is accurate and sufficient for all calves on all farms. Similarly, it is useful to know the exact IgG concentration when measuring the colostrum quality to ensure the actual volume of colostrum being fed to calves is sufficient to avoid FTPI.

STIGA has some advantages compared with RID. STIGA is faster, requiring only 90 min or less as opposed to 24–48 h required for RID [11], is less laborious, and is suitable for automatization. Furthermore, since all STIGA reagents are produced by a recombinant expression system (*E. coli*), STIGA eliminates the need to use animals for production of reagents (polyclonal antisera), which are necessary for RID [11]. This makes STIGA less expensive than RID and more reliable since the variation between different batches of polyclonal antisera produced can be reduced to a minimum.

Glucose is naturally present in both bovine colostrum and in serum. In colostrum, free glucose can be found only in traces and around 2.9% as a part of lactose [26]. In serum of newborn calves, glucose levels can be as high as 7 mmol/L [27]. Still samples are diluted prior to STIGA detection and this step brings free glucose concentration under the detection threshold while the glucose as part of lactose or other compounds cannot be detected by this assay due to the substrate specificity of trehalase.

A limitation of this study was the use of opportunistic samples. The quality of samples (e.g. storage of the samples or freeze and thaw cycles) could have impacted the performance of the test as has been shown in other studies [28]. This concern was addressed in the blinded study where STIGA was tested on fresh samples and showed much better performance, indicating that STIGA could be more efficient than the other assays in diagnosing poor quality colostrum or FTPI.

Although STIGA was performed in a laboratory setting for this study, the performance of STIGA was examined in a user-friendly format that could be optimized for field-testing (STIGA^**FIELD**^). Strong correlations with RID were achieved with STIGA^**FIELD**^, which uses pre-assembled freeze-dried reagents and a commercially available handheld glucometer for detection of the output signal. This format does not require any assembly of test, but rather only application of the diluted sample; hence, it eliminates the need for laboratory equipment and specifically trained personnel.

## Conclusions

We demonstrated that STIGA is as efficient in detecting IgG levels as other devices on the market while having the advantage of being a single step protocol applicable to different sample types (colostrum and serum). The unique feature of this test to directly detect IgG and to produce an easily measurable output signal that makes this test a promising precursor for future field testing.

## Abbreviations

FTPI: Failure of transfer of passive immunity
IgG: Immunoglobulins G
IM: Immunoassays
IR: Infrared spectroscopy
NPD: Negative predictive value
OD: Optical density
PPD: Positive predictive value
RID: Radial immunodiffusion
STIGA: Split Trehalase IgG quantification assay
